# Novel Surfaces in Extracorporeal Membrane Oxygenation Circuits

**DOI:** 10.3389/fmed.2018.00321

**Published:** 2018-11-20

**Authors:** Andrea Ontaneda, Gail M. Annich

**Affiliations:** Department of Critical Care Medicine, The Hospital for Sick Children, University of Toronto, Toronto, ON, Canada

**Keywords:** extracorporeal circuit, anticoagulation, blood-surface interaction, surface coating, nitric oxide, endothelium

## Abstract

The balance between systemic anticoagulation and clotting is challenging. In normal hemostasis, the endothelium regulates the balance between anticoagulant and prothrombotic systems. It becomes particularly more challenging to maintain this physiologic hemostasis when we are faced with extracorporeal life support therapies, where blood is continuously in contact with a foreign extracorporeal circuit surface predisposing a prothrombotic state. The blood-surface interaction during extracorporeal life support therapies requires the use of systemic anticoagulation to decrease the risk of clotting. Unfractionated heparin is the most common anticoagulant agent widely used in this setting. New trends include the use of direct thrombin inhibitor agents for systemic anticoagulation; and surface modifications that aim to overcome the blood-biomaterial surface interaction by modifying the hydrophilicity or hydrophobicity of the polymer surface; and coating the circuit with substances that will mimic the endothelium or anti-thrombotic agents. To improve hemocompatibility in an extracorporeal circuit, replication of the anti-thrombotic and anti-inflammatory properties of the endothelium is ideal. Surface modifications can be classified into three major groups: biomimetic surfaces (heparin, nitric oxide, and direct thrombin inhibitors); biopassive surfaces [phosphorylcholine, albumin, and poly- 2-methoxyethylacrylate]; and endothelialization of blood contacting surface. The focus of this paper will be to review both present and future novel surface modifications that can obviate the need for systemic anticoagulation during extracorporeal life support therapies.

## Introduction

The balance between systemic anticoagulation and clotting is challenging. In normal hemostasis, the endothelium regulates the balance between anticoagulant and prothrombotic systems (1). It becomes particularly more challenging to maintain this physiologic hemostasis when we are faced with extracorporeal life support (ECLS) therapies, where blood is continuously in contact with a foreign extracorporeal circuit (ECC) surface predisposing a prothrombotic state (2). The blood-surface interaction during ECLS requires the use of systemic anticoagulation to decrease the risk of clotting. Unfractionated heparin (UNFH) is the most common anticoagulant agent widely used in ECLS, and its effect depends on adequate amount of antithrombin (AT). Only 1/3 of the dose given can bind to AT, forming AT/UNFH complexes that irreversibly inhibits thrombin activation, factor X activation, and to a lower degree factors IX, XI, and XII activation. It is important to note that the complex is not effective in pre-existing clots, as thrombin that is bound to fibrin will not be inhibited by it. The advantages of using UNFH are its short half-life, easy bedside titration and reversibility by protamine. Despite these advantages it poses a range of complications that increase morbidity and mortality in the ECLS population, including the risk of developing heparin-induced thrombocytopenia (HIT). New trends in ECLS include the use of direct thrombin inhibitor (DTI) agents for systemic anticoagulation (3, 4); and surface modifications that aim to overcome the blood-biomaterial surface interaction by modifying the hydrophilicity or hydrophobicity of the polymer surface; and coating the circuit with substances that will mimic the endothelium or anti-thrombotic agents (4).

The focus of this paper will be to review both present and future novel surface modifications that can obviate the need for systemic anticoagulation during ECLS.

## Extracorporeal circuitry

The ECC consists of a pump, a membrane oxygenator, polyvinyl chloride (PVC) tubing and connectors made up of various materials including polycarbonate and polystyrene. Deoxygenated blood is drained by the pump into the membrane oxygenator (MO), and then the oxygenated blood is returned to the patient [depending on the mode of ECMO–venovenous (VV) or venoarterial (VA)]–into the venous or arterial circulation respectively. In both cases, blood circulates outside the body and comes in contact with a large surface area of foreign materials that do not possess endothelial-like properties; ultimately plasma protein adsorption, coagulation and complement activation, and platelet and leukocytes activation and adhesion occur(5). Turbulent flow and shear forces also contribute to thrombus formation. High shear forces will result in higher platelet deposition and lower fibrin deposition (6, 7), and can induce platelet aggregation (8). Turbulent flow results in hemolysis and cellular activation and this occurs in areas within the ECC that narrow or expand usually within areas of connection and transition to different components of the circuit. Unfortunately, the mechanisms contributing to thrombus formation are still poorly understood (5).

## Surface modifications

Thrombus formation and blood biomaterial surface interaction has been well-described for several decades (9), with protein adsorption (mainly fibrinogen and albumin) occurring rapidly as the blood contacts the artificial surface (10), leading to platelet adhesion/activation and thrombin activation, resulting in thrombus formation (11). To improve hemocompatibility in an ECC, replication of the anti-thrombotic and anti-inflammatory properties of the endothelium is ideal. Different surface modifications have been designed, and others are still undergoing research and development, not only to target thrombin inhibition (antithrombogenic) but also to inhibit platelet adhesion/activation.

We can classify the different surface modifications into three major groups: biomimetic surfaces [heparin, nitric oxide (NO), and DTI]; biopassive surfaces [phosphorylcholine (PPC), albumin, and poly-2-methoxyethylacrylate (PMEA)]; and endothelialization of blood contacting surfaces (4, 12). A combination of surface passivation and biomimetic surfaces are already in use in clinical practice with heparin coating (13) and zwitterionic PPC polymers (14). This paper will give an overview of the different surface modifications that are currently available, and the ones undergoing research.

## Biomimetic surfaces

### Heparin-bound circuit (HBC)

Gott et al. described the first HBC in 1963, where he found that heparin-coated surfaces with an attachment to a colloidal graphite coating, remained clot free for at least 14 days when compared to not coated ones which formed clots within 2 h (15). Ionic heparin binding technique followed, where heparin binds to artificial surfaces by quaternary ammonium ions, taking advantage of the polyanionic nature of heparin. This technique has the disadvantage of heparin leaching from the artificial surface, and the tendency of the oxygenator surface to swell and occlude (16). HBC coatings using covalent bonding are reported to not have heparin leaching (12, 17).

Extensive research [mostly on cardiopulmonary bypass (CPB) circuits] has been done in the last 50 years demonstrating that HBC can significantly decrease the incidence of blood transfusion requirements; decrease repeat sternotomy, duration of ventilation, intensive care unit (ICU) length of stay (LOS), and hospital LOS (18). Ranucci et al described in his meta-analysis that only a reduction in atrial fibrillation rate and a shorter stay in the ICU remained significantly associated with the use of biocompatible surfaces (19). *In vitro* models (13) reported a decrease in typical inflammatory response initiated by blood contact with artificial surface; and *in vivo* models (20, 21) and clinical studies (22–24) showed that heparin-coated surfaces reduce cellular activation and the release of inflammatory mediators. In a study done by Jansen et al. in 30 patients undergoing CPB, 15 of them were treated with HBC and demonstrated reduced complement activation, improved biocompatibility and postoperative performance (25).

HBC does not obviate the need for systemic anticoagulation during ECLS and therefore the complications and effects of heparin remain (11, 12, 26, 27). Although rare, HIT is one of the most serious complications. In the context of HBC, HIT management can be challenging, as the first-line of treatment is the immediate discontinuation of all heparin sources. It is still unclear whether HBC affects HIT antibody generation as different studies have found conflicting results (28, 29).

### Nitric oxide (NO) biomimetic surfaces

NO is generated and released by endothelial cells (along with prostacyclin) and is an endogenous vasodilator, and an anti-platelet agent with both direct and indirect effects on the platelet that suppress activation and aggregation. NO activates soluble guanylyl cyclase (GC-S) by binding to the hemoprotein of the enzyme. This interaction converts magnesium guanosine 5′-triphosphate to guanosine 3′, 5′-monophosphate (cGMP) leading to rapid increase in intracellular concentrations. cGMP system regulates intraplatelet calcium (Ca 2+) levels, the release of platelet granules, and the activity of platelet receptors. It also regulates the expression of IIb/IIIa fibrinogen receptor on the platelet surface and translocation and release of P-selectin from platelet alpha granules to the platelet membrane (30). Platelet inhibition mediated by NO, in both vascular smooth muscle cells and platelets, is primarily cGMP-dependent, requiring activation of downstream protein kinase G (PKG). NO-mediated cGMP-independent platelet adhesion inhibition can happen in response to exogenous NO by S-nitrosylation of platelet proteins (31).

These mechanisms will temporarily inhibit platelets, and the effect lasts milliseconds as NO is scavenged by different hemoproteins due to the high binding affinity for heme iron (11). Thereafter, once platelets are not exposed to NO, they recover their normal function. NO also inhibits bacterial proliferation and adhesion.

NO has been studied extensively for incorporation into polymers. A comprehensive review of the use of NO to prevent thrombosis in ECC has been done by Reynolds et al. (11). Two different methods in which NO can inhibit platelets during ECLS were described: the first method infuses NO into the gas of the membrane oxygenator; and the second method incorporates NO into the polymers of the circuit in a controlled manner for local release at only the blood biomaterial interface with no effect systemically. They further divide the NO-releasing ECC into three generations: In the first generation, an NO compound (MAHMA/NO) was added into a thin layer of plasticized PVC creating a linear NO surface flux and compared in 4 different groups of animals in a 4 h ECC run, demonstrating reduced platelet consumption and adhesion in the NO-doped-surface compared to controls, and no benefit to simultaneous systemic heparin use. Despite the anti-thrombogenic properties of this surface, leaching of the entire NO compound causing release of not only NO at the surface of the circuit but also systemic release of the nitrosamine. Second generation studies involved silica-based NO-releasing materials (DACA-SR/NO and Silica/NO) in a non-heparinized rabbit model for 4 h. Despite DACA-SR/NO demonstrating decrease in platelet consumption and no leaching of compounds, it did not achieve the desired time to release of NO, and the required thickening of the coating to provide an adequate reservoir of NO release over time resulted in circuitry that was no practical for clinical use. Silica/NO particles were also designed, for incorporation into polymer matrices that were applied to the ECC. This modification prevented leaching, allowed NO surface flux variations, and also showed reduction in platelet consumption and activation; however in a swine model of ECC using the same Silica/NO surface for 24 h, the raceway of the circuit was easily delaminated and the NO reservoir was depleted over the 24 h causing platelet consumption. The third generation of NO releasing ECCs involved embedding a lipophilic NO donor complex (DBHD/NO) into plasticized PVC to overcome the previous generations' pitfalls. This NO compound allowed close control of NO release, did not leach and maintained durability. Platelet consumption was proportional to NO release until the optimal level was achieved. Above the optimal flux there was no added benefit to decrease platelet consumption nor increase platelet inhibition. All NO-doped-surfaces maintained platelet function while preventing platelet consumption, with no systemically significant methemoglobin generation observed.

Exposure of blood to foreign material will cause a series of events that as explained above starts with plasma protein adsorption, mainly fibrinogen. Fibrinogen once adsorbed, becomes denatured and exposes its receptor binding sites leading to subsequent adhesion of activated platelets. Fibrinogen converts to insoluble fibrin, which ultimately forms a thrombus. The third generation NO compounds although demonstrating excellent platelet inhibition and maintenance of hemostasis, did demonstrate fibrinogen consumption (32, 33). A multi-functional bilayer polymeric coating with an NO-donor, heparin and thrombomodulin developed by Wu et al. demonstrated prevention of thrombosis in a rabbit model of ECC (34). The DTI, argatroban was incorporated into the extracorporeal circuits, and after 4 h significantly less thrombus formation was evidenced in this group. Leaching of the DTI was observed in the *in vitro* model, however in the *in vivo* model it did not result in systemic levels of anticoagulation (35). In addition there was also conservation of fibrinogen levels. Most certainly the above studies and others that carried out similar covalent modification strategies by incorporating the NO into the polymer backbone of ECC surfaces have also demonstrated proof of principal that local NO release at the blood biomaterial surface interface reduces platelet consumption and eliminates the need for systemic heparinization (36), and reduces platelet activation and thrombus formation, while preserving primary hemostatic function (32, 37) within the body.

Protein adsorption to artificial surfaces is not exclusive to hemostatic proteins, bacterial adhesion also occurs and results in biofilm formation on the artificial surface which elevates the risk for device-related infections. A mature biofilm will release bacteria from the biofilm colony periodically, and is a physicochemical barrier to antibiotics. Different strategies for creating antibacterial surfaces have been developed and include surfaces that resist bacteria and reduce initial attachment, surfaces that detach biofilms, and surfaces that have bactericidal functions (38). And although not the scope of this paper, in review of several studies looking at NO polymers, it has been well-described that NO appears to have both bactericidal effects, and the capability to prevent biofilm formation.

The ideal artificial surface should therefore have antiplatelet and antithrombin properties to target platelet adhesion and fibrin adhesion, in addition to antibacterial properties. Such NO releasing surfaces described above demonstrate all three properties.

Despite these impressive qualities, NO releasing surfaces have limitations and these are most problematic in the very long run ECMO course. The main limitation is that there is a fixed time of release due to a finite NO donation reservoir, which is depleted after about 4 weeks. In addition, the unique NO releasing properties are destroyed at high temperatures and therefore regular manufacturing techniques for tubing extrusion cannot be used. Thus, new techniques such as a dip-coating method need to be developed thus requiring partnership between the researcher and the manufacturer.

Alternative to the NO releasing surface with a finite NO source vulnerable to heat extrusion, other strategies for local NO release at the blood biomaterial surface have also been pursued. These involve building endogenous NO reservoirs from NO donors [S-nitrosothiols (RSNOs) and N-diazeniumdiolates (NONOates)] and using them as alternative biomimetic surfaces. This approach has been studied over the years. NONOates species are a solid, stable compound. The rate of NO release can be closely controlled from seconds to days depending upon the chemical structure and amount of amine precursors. RSNOs are synthesized by reacting thiols (R–SH) with nitrous acid (HNO 2). Their presence throughout the host tissues and in physiological fluids such as plasma and in the blood acts as a soluble pool of NO offering advantages over NONOates. The most common RSNOs used for *in vivo* pre-clinical studies are *S*-nitroso- *N*-acetylpenicillamine (SNAP) and *S* –nitrosoglutathione (GSNO) (39).

Metal-organic frameworks (MOFs) are part of this alternative biomimetic approach. MOFS are crystalline materials of metal ions combined with organic ligands forming different multidimensional shapes depending on the variation of their structure. This results in different physicochemical characteristics. Several studies have taken place evaluating MOFs as vehicles for drug delivery (40, 41). Copper-based MOFs generate NO from endogenous RSNOs, and when incorporated into polymer material, Neufeld et al. showed local antiplatelet effect by NO generation, and no copper leaching. MOFs surface modification is safe, resists high temperatures so can be sterilized, and active even after exposure to blood (42).

Inclusion of transition metals such as copper or organoselenium compounds into the artificial surface also creates an NO/blood local interface by reacting with RSNOs in physiological fluids and catalyzing NO production (39). A recent *in vitro* study of a NO-catalytic coating showed maintenance of NO production for 60 days and with local platelet inhibition (43). Few studies have demonstrated that thin polymers prepared with copper particles or copper (II) ligands can also generate NO from S-nitrosothiols *in vitro* (44–46). Copper incorporated directly into polymers causes local continuous release of NO, preventing platelet adhesion and activation, however the risk of copper leaching exists (47).

Nanotechnology such as silica or metallic nanoparticles, hydrogels and nanoliposomes, offer potential improvements in NO donors integration and delivery (48). Carbon nanotubes have been studied as potential use for drug and gene delivery systems, biosensor components and as antimicrobial surfaces. Gaffney et al. tested the biocompatibility of multi-walled carbon.

Nanotubes (MWCNTs), surface-bound to PVC in both an *in vitro* flow model of ECC using human blood and an *in vivo* rabbit flow model of ECC. Unfortunately the results of this surface-modification (PVC with MWCNTs) led to platelet activation and thrombosis (49). Thus, despite a good theoretical consideration, further work/research is required to determine a better means by which to design the MWCNTs so they are non-thrombogenic, as they would be a very efficient method of local NO release at the blood biomaterial surface.

## Biopassive surfaces

### Phosphorylcholine (PPC)

PPC is the predominant hydrophilic polar head group of phospholipids in the outer membrane cell. The asymmetric lipid bilayer membrane has the negatively charged phospholipids in the inner cell membrane, while the zwitterionic phospholipids containing PPC are in the outer membrane. Research has shown that while the negatively charged phospholipids are thrombogenic in nature (50), PPC containing phospholipids are non-thrombogenic (50–52).

PPC coating of ECC has been shown to have a favorable effect on platelets evidenced by a plateau formation of thromboxane B2 and thromboglobulin when compared to the uncoated control ECC group; possibly due to the affinity of PPC coating for phospholipids forming an organized layer on the surface mimicking a biomembrane (53). PPC has also demonstrated less post-operative bleeding and may safely reduce systemic heparinization during CPB (54, 55); and it may also reduce intraoperative thrombin formation (56). However, some studies did not show superiority when compared to HBC (57), or when used in combination (58).

Most PPC coatings are non-crosslinkable amphiphilic copolymers. Crosslinking of a polymer coating can enhance its stability of resisting dissolution or surface reorientation. Wang et al. developed a stable PCC method by crosslink treatment and when comparing it to uncoated ECC surfaces, they showed that protein adsorption, platelet adhesion and activation were suppressed remarkably (14). Another PPC-based copolymer was synthesized by Nagahashi et al. demonstrating significantly reduced protein adsorption with the effect lasting 84 days (59).

### Poly-2-methoxyethylacrylate (PMEA)

PMEA is an amphiphilic polymer with a hydrophobic polyethylene backbone part and a mildly hydrophilic tail. Most of the research has been done in CPB population and has shown reduction of platelet adhesion, platelet aggregation and protein adsorption (60–63). In a swine model, reduced complement activation during CPB was demonstrated (61); and in a clinical study in the pediatric cardiac surgery population, decreased activation of the coagulation system and the inflammatory reaction (64).

When compared to HBC, PMEA coating might be superior to HBC in suppressing the adsorption of plasma proteins such as fibrinogen (61) and might reduce the need for procedural platelet infusions (65). It was equal in preventing reactions induced by CPB circuits (significantly lower levels of thrombin-antithrombin complex and bradykinin) (66). In contrast, other studies report increased incidence of post-procedural leukopenia and possibly systemic inflammatory response syndrome (SIRS) (67), with no difference in platelet aggregation (68), and perhaps inferior to heparin coatings in suppressing complement activation (63).

### Fluid-repellent surfaces (omniphobic)

Development of liquid-repellent microtextured surfaces that rely on the formation of a stable air–liquid interface has been researched for several years and is inspired from the natural non-wetting structures like lotus leaves (69). It has also presented many challenges that have restricted their applications, such as the inability to self-heal after physical damage, high cost, among other problems (70). Wong et al. (70) created a self-healing slippery liquid infused porous surface(s) (SLIPS) with exceptional liquid- and ice-repellency, pressure stability and enhanced optical transparency, using nano/microstructured substrates to lock in place the infused lubricating fluid.

Further recent research has been done by Leslie et al. (71) creating a bioinspired, omniphobic coating for medical devices. Medical grade materials have highly smooth surfaces. In order to create non-adhesive, anti-thrombogenic surfaces for smooth surfaces, they developed an omniphobic surface by modifying the SLIPS technology. Coating was composed of a covalently tethered, flexible molecular layer of perfluorocarbon, which holds a thin liquid film of medical-grade perfluorocarbon on the surface. This was a two-fold study: (1). The *in vitro* portion showed that this coating prevented fibrin attachment, reduced platelet adhesion and activation, suppressed biofilm formation and was stable under blood flow condition. (2). The *in vivo*, animal model used the coating on medical-grade tubing and catheters were assembled into arteriovenous shunts and implanted in pigs and this model demonstrated intact patency of the tubing and catheters for at least 8 h without systemic anticoagulation. This coating technology was able to completely repel blood and suppress biofilm formation while demonstrating the ability to reduce systemic anticoagulation and prevent thrombotic occlusion and biofouling of medical devices.

More recently, Badv et al. reported an efficient, non-invasive process for coating catheters with an antithrombotic, omniphobic lubricant-infused coating produced using chemical vapor deposition (CVD) of hydrophobic fluorine-based organosilanes. When compared with uncoated catheters, CVD coated catheters significantly attenuated thrombosis, and when compared with the commonly used technique of liquid phase deposition (LPD) of fluorine-based organosilanes, the CVD method was more efficient and reproducible, resulting in less disruption of the outer polymeric layer of the catheters and produced greater antithrombotic activity (72).

This is obviously an area of further research, however with such promising results it will likely have a major influence in the next generation of biopassive surface modifications in extracorporeal and also intracorporeal devices.

## Endothelialization

The endothelium regulates the balance between anticoagulant and prothrombotic systems (1), and is characterized by its total compatibility with blood. Quiescent phenotype endothelial cells (ECs) form a monolayer in the endothelium, and with its anticoagulation and anti-proliferation properties creates a non-thrombogenic surface, and prevents smooth muscle cell (SMC) proliferation thereby inhibiting intimal hyperplasia (IH) (73). Research has been directed at two approaches: mimicking the characteristics of the endothelium which has been discussed extensively in the literature; and inducing the endothelialization of the surface itself (74).

Endothelialization of the surface can be achieved by two different methods: (I) *in vitro* pre-endothelialization of the ECC, or (II) endothelial progenitor cells (EPCs)-based *in vivo* induced self-endothelialization (4, 73, 75, 76).

### *In vitro* pre-endothelialization

*In vitro* endothelialization of a biomimetic matrix of adhesive cells and growth factors can improve long term patency and prevent thrombogenesis of grafts (74). *In vitro* endothelialization was initially developed by directly seeding autologous ECs onto the lumen-facing side of synthetic vascular grafts, stents, and tissue-engineered blood vessels before implantation. This has been extensively explored as a technique to inhibit restenosis and thrombus formation. However, there are many drawbacks as the completion of endothelialization of the different types of stents can take from months up to 2 years, and in synthetic vascular grafts there is insufficient EC migration and proliferation to the middle section of the lumen (73). It is also a tenuous process with long culture times and cannot be implemented in emergency cases; poses a risk of contamination and infection, is cost ineffective and is limited to facilities with the ability to do it.

*In vitro* endothelialization by surface modification molecules has been researched during the last two decades (77–79). The interactions between ECs and extracellular matrix (ECM) are primarily mediated by integrins; triggering several intracellular signaling pathways, which regulate EC proliferation, migration, and differentiation (80). Surrounding ECM molecules, such as fibronectin (Fn), have been applied for surface endothelialization modifications, demonstrating that Fn coatings may improve EC adhesion, spreading, proliferation, and migration (77). Despite extensive research in this topic, there are still several limitations to this technique that preclude its use in clinical practice for extracorporeal devices: the matter of the stability of the pre-seeded EC monolayer *in vivo*, prolonged production times, and the need to further study these interactions under different blood flow conditions to evaluate shear force impact.

### *In vivo* induced self-endothelialization

Also known as *in situ* endothelialization, it is an alternative approach to induce rapid self-endothelialization. EPCs are mononuclear cells derived from bone marrow that circulate at low concentrations in peripheral blood and have the potential to differentiate into mature functional ECs. They have the ability to generate functional endothelium *in vivo*, and play an important role in vascular repair and reendothelialization (75).

In order to be successful in *in vivo* induced self-endothelialization, the biomaterial needs to be able to induce EPCs mobilization, homing, migration, and differentiation into ECs (81); and have favorable bioactivity to stimulate *in situ* cell adhesion and proliferation. Surface hydrophilicity, increase in roughness and negative charge characteristics have been reported to enhance EC adhesion and *in situ* migration. Modification of the physicochemical composition of the surface impacts cell differentiation (73, 82). Over the last few years, research has been directed to explore the different strategies to achieve in s*itu* endothelialization. Liu (73) and Pang (75) have published detailed reviews on the several processes researched to improve this technique: cellular and pharmacological therapy directed to increase the concentration of circulating EPCs; and surface modifications with biofunctional molecules such as monoclonal antibodies, nucleic acid aptamers, cytokines and genetic modifiers to induce EPC aggregation, adhesion, and differentiation. Although this is a promising advance in the field and constantly evolving, we still face many limitations and challenges as this is a complex process due to low EC proliferation activity, difficulty in controlling cell behavior; and creating the “ideal” artificial surfaces that will enhance EPCs functional abilities, promote selective adhesion of EPCs and ECs onto its surface and inhibit thrombogenesis at the same time, while combining all of the different strategies.

Recent research in nanofabricated cardiac grafts enhanced with biomaterials that can promote *in situ* endothelialization without intimal hyperplasia and thrombosis occurring during endothelium formation has been taking place (74) but is still in the preliminary stages of development.

## Conclusions

The preservation of hemostasis within the patient while maintaining patency of the extracorporeal circuit remains one of the most challenging aspects of ECLS management. It is becoming very clear that although algorithms and new anticoagulants may make a difference in the bleeding and thrombotic complications associated with ECLS, they do not work the same for each patient and as a result the anticoagulation management for each patient must be customized to them using all that is presently available in terms of monitoring, anticoagulants and surface modifications. This review details the present and future work needed to develop an extracorporeal circuit that can function as the endothelium so that systemic anticoagulation can be obviated. It will likely have some aspect of biomimetic and biopassive properties with a living cellular interface (see Table 1). To design such a surface for clinical use several processes presently used for circuit production will need to be redesigned or in fact be developed from scratch. The biomimetic surfaces described above cannot undergo usual manufacturing production by hot extrusion, therefore development of mass mandrel systems to dip coat each catheter and tubing length to apply the surface need to be developed. Or perhaps there is a potential for a minimal heat extrusion at which tubing can be produced and the biomimetic surface can be preserved. The toxicities of a biopassive surface such as the recent omniphobic surfaces need to be further explored since devices such as ECLS circuits may in fact be used on patients for weeks to months. And finally the advancement in endothelialization is absolutely inspiring, however customization of the circuit to the patient's own endothelial cells is not an undertaking that can be done in a day much less months to a year. This does not remove it as a potential surface but rather limits it to either a specific part of the entire circuit, such as the oxygenator, or to being used in a patient where eventually artificial support may be required. This makes it remain in an elective/chronic illness category for support, not one that ECLS is frequently a part of.

**Table 1:** Current problems and future perspectives of surface modifications.

**Type of surface modification**	**Problems**	**Future perspectives**
Heparin-bonded (HBC)	Ionic binding: heparin leach and tendency of oxygenator to swell and occlude. Does not obviate need for systemic anticoagulation.	HBC using covalent bonding are reported to not have leaching of heparin
Nitric oxide (NO)-releasing	*First generation (MAHMA/NO):* Entire molecule leaching releasing nitrosamines into the blood. Second generation (silica-based): - DACA-SR/NO: Delay in NO release time and required thickening of the coating to provide an adequate reservoir of NO not practical for clinical use. - Silica/NO: NO reservoir depletion over 24 hours due to circuit raceway delamination. *Third generation (DBHD/NO):* fibrinogen consumption. NO releasing properties are destroyed at high temperatures thus impractical for standard tubing production through extrusion. NO releasing surfaces have a finite reservoir which is depleted after about 4 weeks.	Surface modification strategy that avoids NO leaching is successful with DBHD/NO. The molecule remains in the organic phase of the polymer. Addition of topcoat with direct thrombin inhibitor prevents fibrinogen consumption. In addition antibacterial properties of NO will suppress biofilm formation. NO-release is controlled by modulating the pH within the polymer and threshold flux of NO required to inhibit platelet activation can be finessed. While argatroban prevents fibrinogen adhesion/consumption. Alternative method of manufacture either by mandrel dip coating or cold extrusion to retain the biomimetic properties. Thus an NO compound that allows close control of NO release, no leaching and maintains durability. Using endogenous NO reservoirs from NO donors as alternative biomimetic surfaces (metal-organic frameworks; nanotechnology) is an option to a finite reservoir of NO release.
Omniphobic surfaces	Undergoing research with coating for medical devices that needs to yet be tested in extracorporeal circuits.	Develop a non-adhesive, anti-thrombogenic surface for extracorporeal circuits that will suppress biofilm formation, and will reduce the need for systemic anticoagulation.
Endothelialization	In vitro: - Completion of endothelialization can take months to years. - Tenuous process with long culture times and cannot be implemented in emergency cases. - Risk of contamination and infection - Cost ineffective and limited to facilities with the ability to do it. In vivo: - Low endothelial cell proliferation activity.	Create the ideal artificial surface that will enhance endothelial progenitor cells function and adhesion and inhibit thrombogenesis. Customize long term respiratory and cardiac support devices to the patient by seeding the devices with the patient's endothelial cells. Would obviate the need for aggressive anticoagulation if any.

Until this ideal surface can be designed it is inspiring to see that even further development within one of the three categories of surface modifications can likely already make a major difference in the bleeding/thrombotic complications of ECLS. The time to bring industry and bench research together is now. The need for such a surface is now, as we continue to place higher and higher risk patients on ECLS for longer and longer times.

## Author contributions

AO wrote the manuscript draft and finalized changes after revision and approval. GA manuscript revision, read, and approved the submitted version.

### Conflict of interest statement

The authors declare that the research was conducted in the absence of any commercial or financial relationships that could be construed as a potential conflict of interest.
